# Sensbio: an online server for biosensor design

**DOI:** 10.1186/s12859-023-05201-7

**Published:** 2023-02-28

**Authors:** Jonathan Tellechea-Luzardo, Hèctor Martín Lázaro, Raúl Moreno López, Pablo Carbonell

**Affiliations:** 1 Institute of Industrial Control Systems and Computing (AI2), Universitat Politècnica de València (UPV), 46022 Valencia, Spain; 2 Institute for Integrative Systems Biology I2SysBio, Universitat de València-CSIC, 46980 Paterna, Spain

**Keywords:** Biosensor, Transcription factor, Synthetic biology

## Abstract

Allosteric transcription factor (aTF) based biosensors can be used to engineer genetic circuits for a wide range of applications. The literature and online databases contain hundreds of experimentally validated molecule-TF pairs; however, the knowledge is scattered and often incomplete. Additionally, compared to the number of compounds that can be produced in living systems, those with known associated TF-compound interactions are low. For these reasons, new tools that help researchers find new possible TF-ligand pairs are called for. In this work, we present Sensbio, a computational tool that through similarity comparison against a TF-ligand reference database, is able to identify putative transcription factors that can be activated by a given input molecule. In addition to the collection of algorithms, an online application has also been developed, together with a predictive model created to find new possible matches based on machine learning.

**Supplementary Information:**

The online version contains supplementary material available at 10.1186/s12859-023-05201-7.

## Introduction

Biosensors allow researchers from various fields to use biological systems to detect external or internal signals and to react to those signals in a designed manner [1]. Among other inputs, biosensors can be used to detect small molecules that may play important roles in areas such as bioremediation, metabolic engineering, or biocomputing. An important class of biosensors is the one based on allosteric transcription factors (aTFs) that bind to the molecule, triggering the expression or repression of a particular gene (e.g., a reporter gene). Even though biosensors have been used for a wide range of applications, the number of known responsive TFs is still limited compared to the number of potential chemical targets of interest in many applications.

During recent years, both computer and experimental assays have been reported in the literature describing different methods to discover new TF-ligand interactions, including bioprospecting and metagenomics. However, such multi-step process may, collectively, involve years of research. The efforts required to find a new TF from the available genomic knowledge, characterize it properly, and validate its functionality against a new molecule presents a high toll to pay for the biosensor designer. For these reasons, more computational databases and tools are needed to help in the design of new biosensors, especially in the prototype phase. For example, Sensipath [2] is specialized in finding the closest detectable compounds connected through metabolic pathways to the query compound. Basically enabling the use of indirect sensing when the query has not known TFs that can be used to measure it directly. Other tools like DeepTFactor [3] try to fill the gap of known TFs by using AI to discover new TFs by other means than homology-based prediction.

Here we present Sensbio, a set of easy-to-use Python algorithms and notebooks and a web application that find new possible TF-ligand interactions by protein sequence and molecular similarity analysis that can be additionally assisted by machine learning-based recommendations. The Sensbio open-source toolbox provides a set of tools to help in the design of transcription-factor based biosensor circuits. Based on a dataset containing 451 chemical compounds and 3507 transcription factor sequences, Sensbio assists synthetic biologists by suggesting potential new TF-ligand interactions based on six different sources of transcription factor data, finding similar molecules and candidate transcription factors to the inputs. Compared to other tools and databases, Sensbio collates the information from the available databases simplifying the research task for the users. It also provides a molecular-string (SMILES) based searching algorithm, thus removing the confusion often found using the molecule's common name making the search of similar compounds unambiguous. Finally, the result of the molecular tool provides a similarity score. Previous databases/tools lacked this feature. With Sensbio, similar alternative compounds to the user’s query are suggested as starting points for biosensor design. In that way, Sensbio allows users to identify existing and novel transcription factor-based biosensors for applications ranging from genetic circuits design, screening, production, and bioremediation of chemicals to diagnostics.

## Material and methods

### Databases, packages and tools used in this study

The dataset published by Koch et al. [4] was used as a starting point for the Sensbio database. It contains a 2018 collection of TF-ligand interactions from different databases and literary resources. To expand and update this dataset, data dumps detailing aTFs and their triggering compounds were collected, cleaned and formatted accordingly from the following databases: BioNemo [5], RegulonDB [6], RegPrecise [7], RegTransBase [8], Sigmol [9] and GroovDB [10].

Custom Python 3 scripts (using standard libraries like Pandas and Numpy) were used to populate, clean, format and analyze the database and to build a web application through the Streamlit framework (https://streamlit.io/). Molecular fingerprints were extracted, analyzed and compared using the RDKit python library [11]. Networkx python module was used to describe and produce the molecular network. A local BLAST+ installation allowed the scoring and ranking of the protein sequences. Ete3 python toolkit [12] produced the phylogenetic trees of the TF sequences. Deep learning techniques were applied to build the predictive model through the Tensorflow and Keras Python libraries.

Classyfire [13] and iFragment [14] external web applications were used to classify the different molecules by chemical and metabolic categories respectively. Classyfire produces a hierarchical list of ontologies. In this case, the parent ontology was kept as the representative category for each molecule. iFragment on the other hand, produces a list of KEGG [15] metabolic pathways ordered by the probability of the input compound to belong to that particular pathway. The three pathways with the lowest *p*-value were selected. Using the KEGG restful API (https://www.kegg.jp/kegg/rest/keggapi.html), the parent ontology was extracted for each pathway and assigned as the final metabolic category.

### Implementation

First, the Sensbio database was built detailing both molecular (molecule common name, SMILES, InChI and information on the metabolic paths where the molecule plays a role) and protein sequence information (TF name, origin species, protein sequence, NCBI and Uniprot accession numbers and database and literature references) for each of the TF-ligand pairs mined from the previously detailed databases and bibliographic sources.

The toolbox built around the database can be used both for searching for novel TF-molecule interactions, and to analyze the state-of-the-art of the aTF-mediated biosensing space. Sensbio accepts protein sequences and chemical compounds as inputs. Two possible use cases for the tool are envisioned:

#### Molecular search: use case 1

**Fig. 1:**
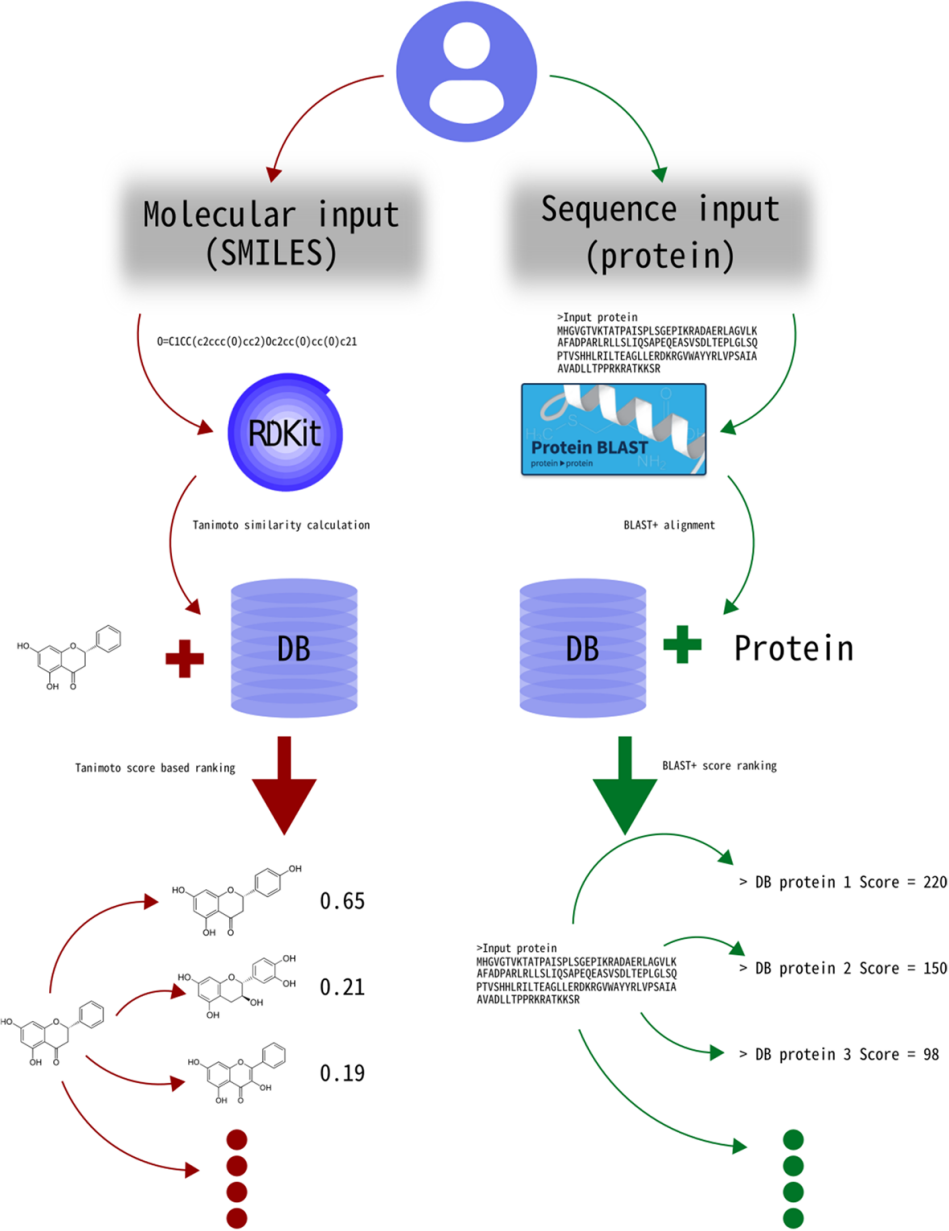
Sensbio workflows. Red flow: a molecular input by the user produces an ordered rank of similar molecules paired with the aTF that is activated or repressed by them. Green flow: a protein sequence input produces a ranked list of sequences and their binding molecule

When the user wants to determine if a chemical compound can be sensed using TF-based biosensors they can use the molecular similarity tool (“molecule” script / notebook) (Fig. 1, red flow). The tool calculates the Tanimoto distance (using the RDkit library) of the input molecule against all the molecules in the database one by one. First, Morgan fingerprints are calculated for the query molecule and the database molecule. This fingerprint is similar to ECFP (Extended-Connectivity Fingerprints) [16] which is one of the most common algorithms for general chemoinformatics purposes.

Then, the Tanimoto similarity score is calculated from both fingerprints. This metric was chosen for several reasons. Firstly, Tanimoto score and other similar metrics were compared for molecular similarity tasks using ECFP and has been proven to be a good metric for this task in previous works [17]. In addition, experimental works showing that alternative ligand molecules can trigger similar TF-mediated gene regulation used Tanimoto as the metric to find the best alternative molecule to the known activating ligand [18, 19].

Once all the Tanimoto distances have been calculated, the tool outputs a rank of the entries in the database linked to each of the molecules (including score, paired molecule, TF sequence, and the remaining information of the entry).

#### Sequence search: use case 2

When the user wants to check a predicted putative TF for sensing capabilities they can use the sequence similarity tool (“sequence” script / notebook) (Fig. 1, green flow). The tool uses the user’s input as a query and BLAST + as alignment algorithm, and the TF sequences as BLAST database. It queries the user protein input against the TF sequences dataset and it provides the top significant set of entries in the database closest to the input sequence as output. This can be used to determine possible molecular ligands and to fast-track a literature search on the closest transcription factors for the query protein.

The repository containing the tool files and requirements is available at: https://github.com/jonathan-tellechea/sensbio.

#### Predictive model

Moreover, a predictive system has been developed with the aim of having a machine-learning based recommendation system for finding new possible TF-ligand interactions. In order to train the model, the Sensbio database was initially used. For the TF sequences, the one-hot encoding technique was used. For the molecules, fingerprints from SMILES were extracted. Also, negative cases, i.e., cases where there is no affinity between the TF and the molecule were generated. For this purpose, a molecule that does not resemble the molecule associated with a given TF based on their Tanimoto index was randomly selected for each sequence.

**Fig. 2:**
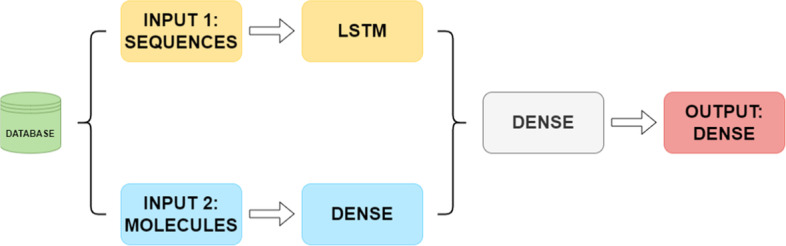
Network architecture diagram

The network architecture (Fig. 2) is based on two branches (one for each type of input), which are then concatenated. For the TF branch a LSTM (long-short term memory) layer was considered as it can learn from sequential data [20]. The optimizer used for the model is the Adam algorithm, and the activation function for both neuron type is ReLU. The hyperparameters were optimized using Bayesian optimization. These parameters were the learning rate, the batch size, and the number or epochs.

In terms of model training, a cross-validation was carried out to test the different possibilities of the hyperparameters of the model. Clustering was used to group the data in the different training and validation set. Each cluster is made following the dissimilarity between the molecules to ensure that the data is evenly split in terms of similarity. The model returns a score between 0 and 1, where a value close to 0 indicates that there is no affinity between the TF and the effector molecule, and a value close to 1 indicates that there exists a potential interaction between both. The repository containing the codes required to build and train this model is available at: https://github.com/pablocarb/biosensor_predictor.

In order to verify the performance of our model, several comparisons have been made with other classifiers. They are the following: SVM, Random Forest and Gaussian Naive Bayes classifier. Moreover, the 1-hot encoding technique was compared with a higher-dimensional embedding representation of the protein sequence using the Embedding layer available in Keras.

## Results

### Molecular similarity

Next, we describe the results of the molecular similarity tool. For this purpose, naringenin (O=C1CC(c2ccc(O)cc2)Oc2cc(O)cc(O)c21) and pinocembrin (C1C(OC2=CC(=CC(=C2C1=O)O)O)C3=CC=CC=C3) molecules are used as examples. When naringenin is fed into the chemical tool, it produces the dataset shown in Table 1. The Tanimoto score of 1 for the first 5 entries confirms that the database contains the target molecule and provides information on 5 TFs that have been described to be activated by this compound. This result informs the user that the input molecule has been described as the activator of these TFs so they can make a decision on their following experimental workflow.

**Table 1:** Sensbio molecular results examples

Input	Molecule	Species	TF	NCBI Accession	Sequence	Tanimoto score
Naringenin	Naringenin	*Sinorhizobium meliloti*	nodD1	WP_010967456	MRFRGLDLNLLVALD…	1
	Naringenin	*Herbaspirillum seropedicae*	fdeR	WP_013233032	MRFNKLDLNLLVALD…	1
	Naringenin	*Pseudomonas putida*	TtgR	BAN52789.1	MVRRTKEEAQETRA…	1
	Naringenin	*Azorhizobium caulinodans ORS571*	nodD	CAA88827.1	MRFKGLDLNLLVALN…	1
	Naringenin	*Azorhizobium caulinodans*	nodD	WP_012172315.1	MRFKGLDLNLLVALN…	1
Pinocembrin	Naringenin	*Sinorhizobium meliloti*	nodD1	WP_010967456	MRFRGLDLNLLVALD…	0.648
	Naringenin	*R. leguminosarum*	nodD	WP_207159894.1	MRFKGLDLNLLVALD…	0.648
	Naringenin	*Azorhizobium caulinodans*	nodD	WP_012172315.1	MRFKGLDLNLLVALN…	0.648
	Naringenin	*Azorhizobium caulinodans ORS571*	nodD	CAA88827.1	MRFKGLDLNLLVALN…	0.648
	Naringenin	*Pseudomonas putida*	TtgR	BAN52789.1	MVRRTKEEAQETRA…	0.648

When a molecule that is not in the database is provided as input, the tool provides the set of entries ordered by Tanimoto score. In the case of the pinocembrin, the application ranks naringenin as the highest entries by close similarity to the compound, suggesting that pinocembrin could be sensed though naringenin-activated TFs. This was experimentally confirmed in Trabelsi et al. [18]. This information could be used by the user to find TFs that are likely to sense their input compound and build prototype biosensor circuits around this information.

The complete results of these two examples and other three example molecules that were not present in the original database can be found in the Additional file 1.

### Sequence similarity

Here we showcase the behavior of the tool when using its sequence similarity feature. Given a TF sequence that is present in the database (e.g. AseR, *B. subtilis*, NP_388414.1 which is triggered by arsenite) the algorithms produce the ranked entries shown in Table 2 (a summarized view of the whole output data). In essence, the software recognizes the sequence as present in the database by giving it the highest rank (based on the BLAST+ scoring system) and 100% identity score. It also provides the user with other relevant sequences that recognize the same compound that may be worth studying further for increased biosensor design space in the laboratory.

**Table 2:** Sensbio example sequence results

Input	Molecule	Species	TF	NCBI Accession	Sequence	Identity %	e-value	bit-score
AseR, *B. subtilis*, NP_388414.1	Arsenite	*Bacillus subtilis subsp. subtilis str. 168*	AseR	NP_388414.1	MTIDVAAMTRCLK…	100	1.91E−82	233
	Arsenite	*Bacillus clausii KSM-K16*	AseR	YP_174335.1	MGFKSLSSEEIAT…	50.485	8.61E−38	120
	Arsenite	*Bacillus amyloliquefaciens subsp. plantarum str. FZB42*	AseR	YP_001423145.1	MERRHHALSSEGI…	54.286	4.29E−36	116
	Arsenite	*Bacillus halodurans C-125*	AseR	NP_243866.1	MASVKQQLEVAT…	42.593	4.76E−32	105
	Arsenite	*Geobacillus kaustophilus HTA426*	AseR	YP_146440.1	MQKTVVEIEKASH…	47.059	2.38E−31	103
ArsR, *Micromonospora maris*	Arsenite	*Nocardia farcinica IFM 10152*	ArsR	YP_118660.1	MSNPSLPVAPVD…	48.718	2.56e−31	104
	Cadmium(cd2+)	*Nocardia farcinica IFM 10152*	ArsR	YP_118660.1	MSNPSLPVAPVD…	48.718	2.56e−31	104
	Silver(ag+)	*Nocardia farcinica IFM 10152*	ArsR	YP_118660.1	MSNPSLPVAPVD…	48.718	2.56e−31	104
	Arsenate	*Nocardia farcinica IFM 10152*	ArsR	YP_118660.1	MSNPSLPVAPVD…	48.718	2.56e−31	104
	Arsenite	*Nocardia farcinica IFM 10152*	SahR	YP_122073.1	MSKSKLVVTPVQA…	50.515	3.39e−30	102

When a TF that is not present in the database is fed to the sequence similarity tool (e.g. ArsR, *Micromonospora maris,* WP_043720559.1) one should expect the results shown in the lower half of Table 2. Again, the script returns a list of the most similar proteins in the database together with information on the species and triggering molecules. This information could be used after discovering a new TF to assess possible molecular targets, together with other sources of information before experimental validation.

### Database analysis

Finally, we highlight in this section the most important features of the Sensbio database, which was collected from several sources as previously described. It contains 451 unique molecules and 3507 protein sequences which interact among themselves producing 5387 unique TF-ligand pairs.

**Fig. 3:**
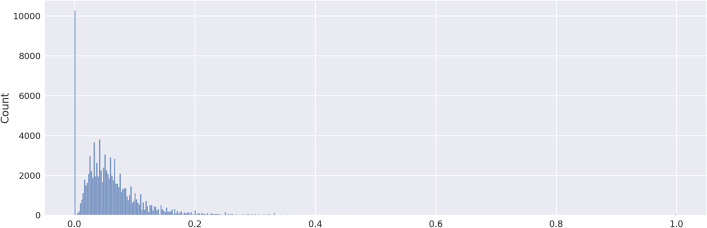
Tanimoto score distribution of the whole molecular collection (451 molecules)

Using the RDkit python library, the Tanimoto score of all the molecules against each other was calculated. For all the possible pairs, the score was stored and plotted in Fig. 3. This figure shows that most of the molecule pairs have a similarity score between 0 and 0.2.

**Fig. 4:**
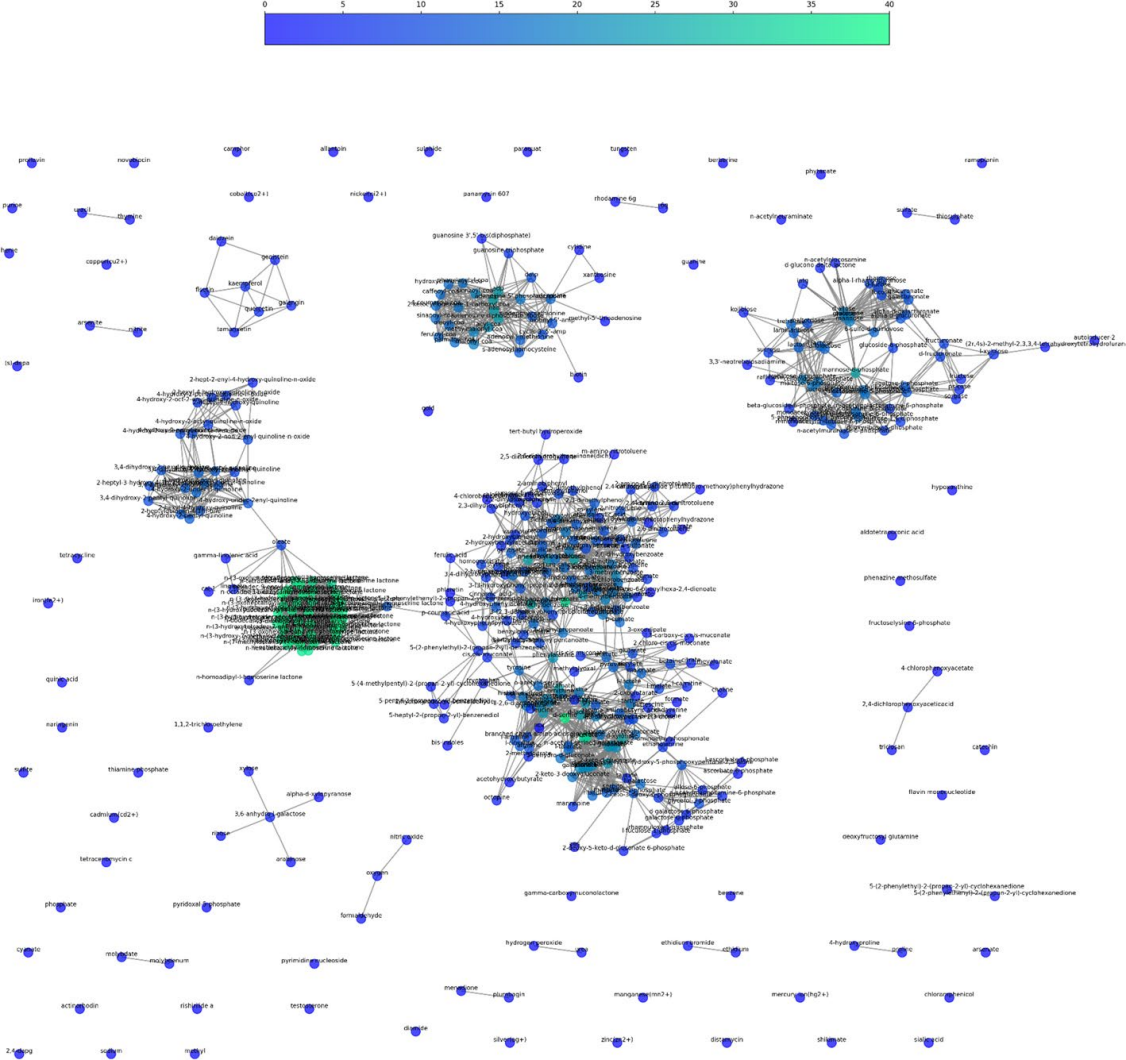
Molecular network and clustering of the database. Two molecules are connected together if they have a Tanimoto similarity score higher than 0.25. The color of the node represents the number of connections of that node

Further analysis using the network python library NetworkX shows how the molecules are related and clustered together by the similarity score (Fig. 4). The network figure groups the molecules in 5 molecule clusters pertaining to similar molecular families (e.g. sugars, quorum sensing).

**Fig. 5:**
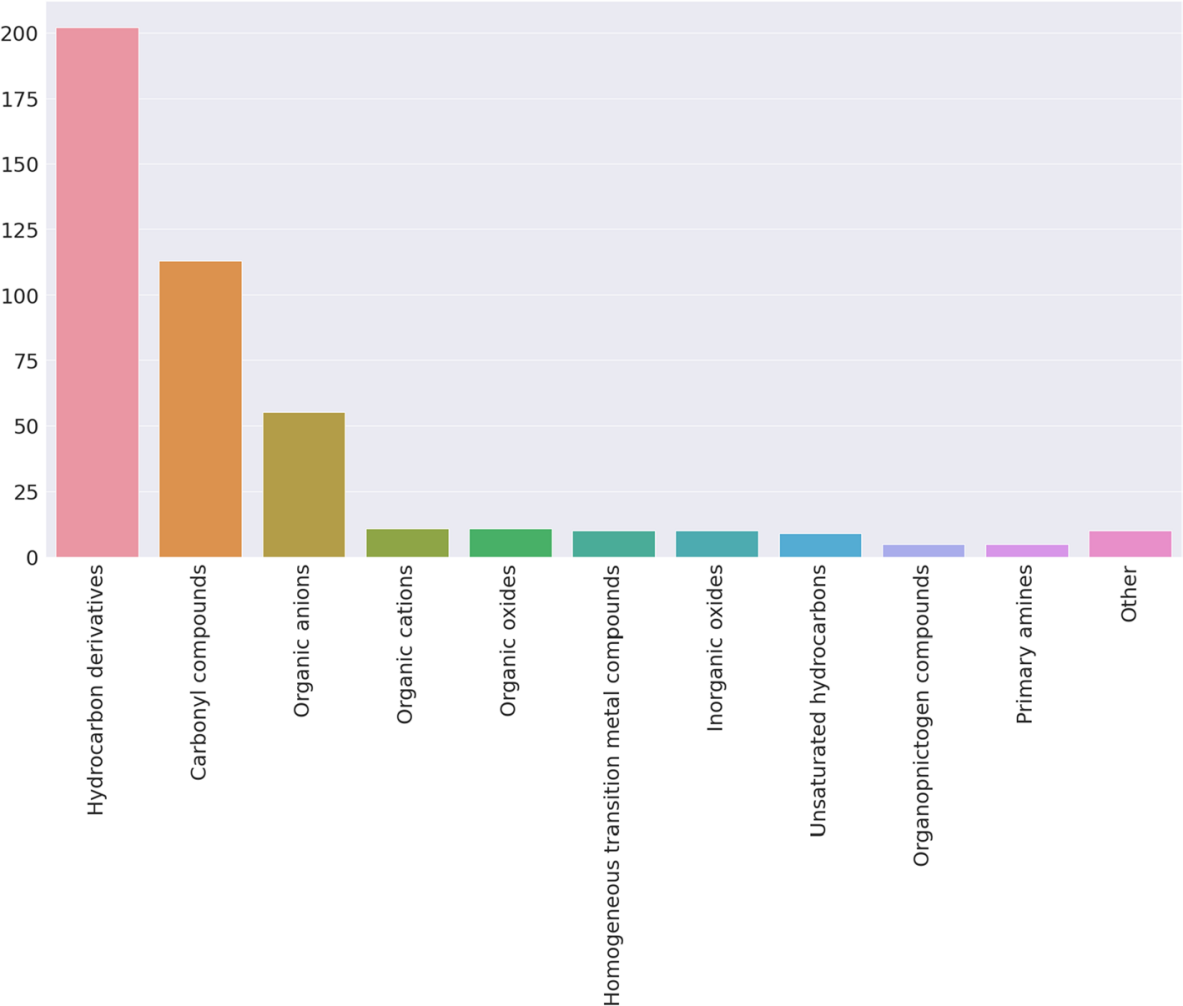
Frequency of molecular ontologies discovered in the database. A total of 25 molecular categories are present

The molecules can be classified using different criteria. First, molecules were classified using chemical ontologies using the Classyfire tool. Figure 5 shows the different chemical categories present in the database and their abundance. The most common category was established as “Hydrocarbon derivatives” (simple and complex sugars, etc.), followed by “Carbonyl compounds” (some amino acids, lactones, etc.).

**Fig. 6:**
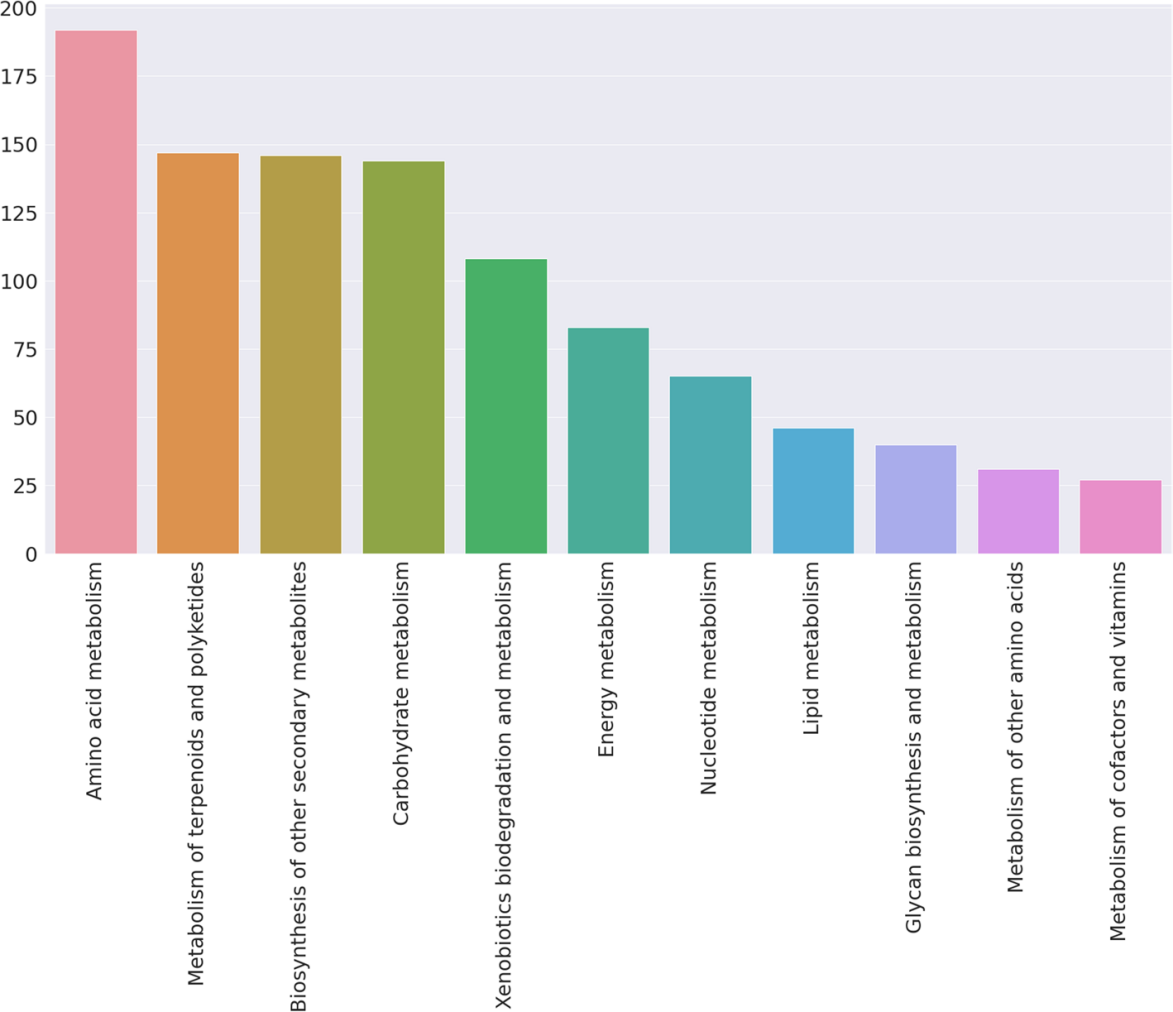
Frequency of metabolic categories found in the database

Another classification can be made using metabolism as main criteria. The iFragment tool was used to assign biological pathways to each of the compounds in the database searching against the KEGG pathways dataset. Figure 6 shows the distribution of different KEGG pathways found. Note that the three most likely KEGG functions assigned to each compound were kept. Most of the molecules in the dataset are related to amino acid metabolism.

**Fig. 7:**
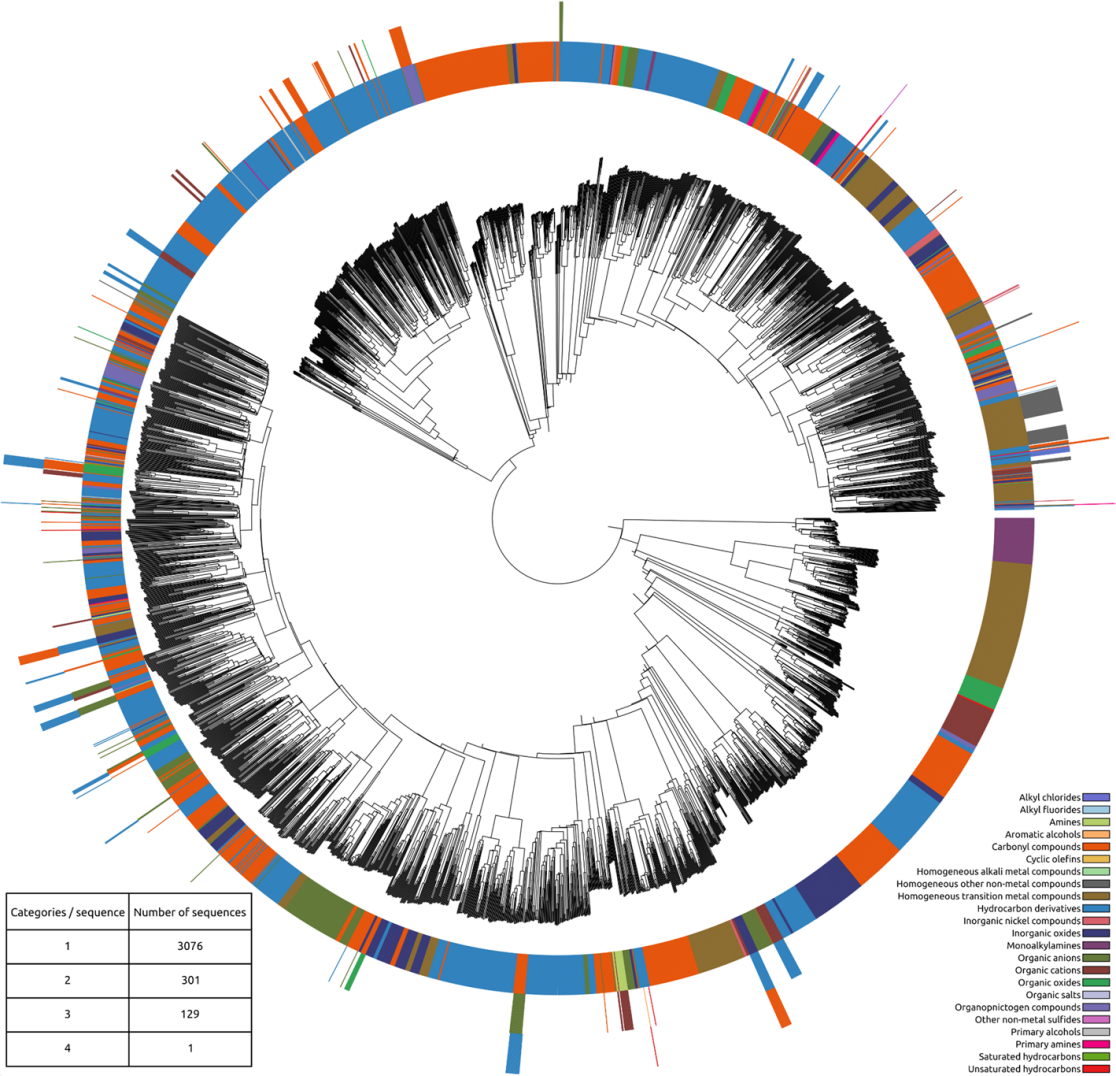
Phylogenetic tree of the protein sequences in the database paired with the chemical categories of the compound(s) that bind to the TF

The protein sequences in the database were analyzed by their relationship with their compound pair. The table in Fig. 7 details how the sequences are related to the chemical categories.

87.7% of the 3507 unique sequences have been associated to a single molecular ontology. The rest are "promiscuous" TFs and are triggered by more than one molecular category.

**Fig. 8:**
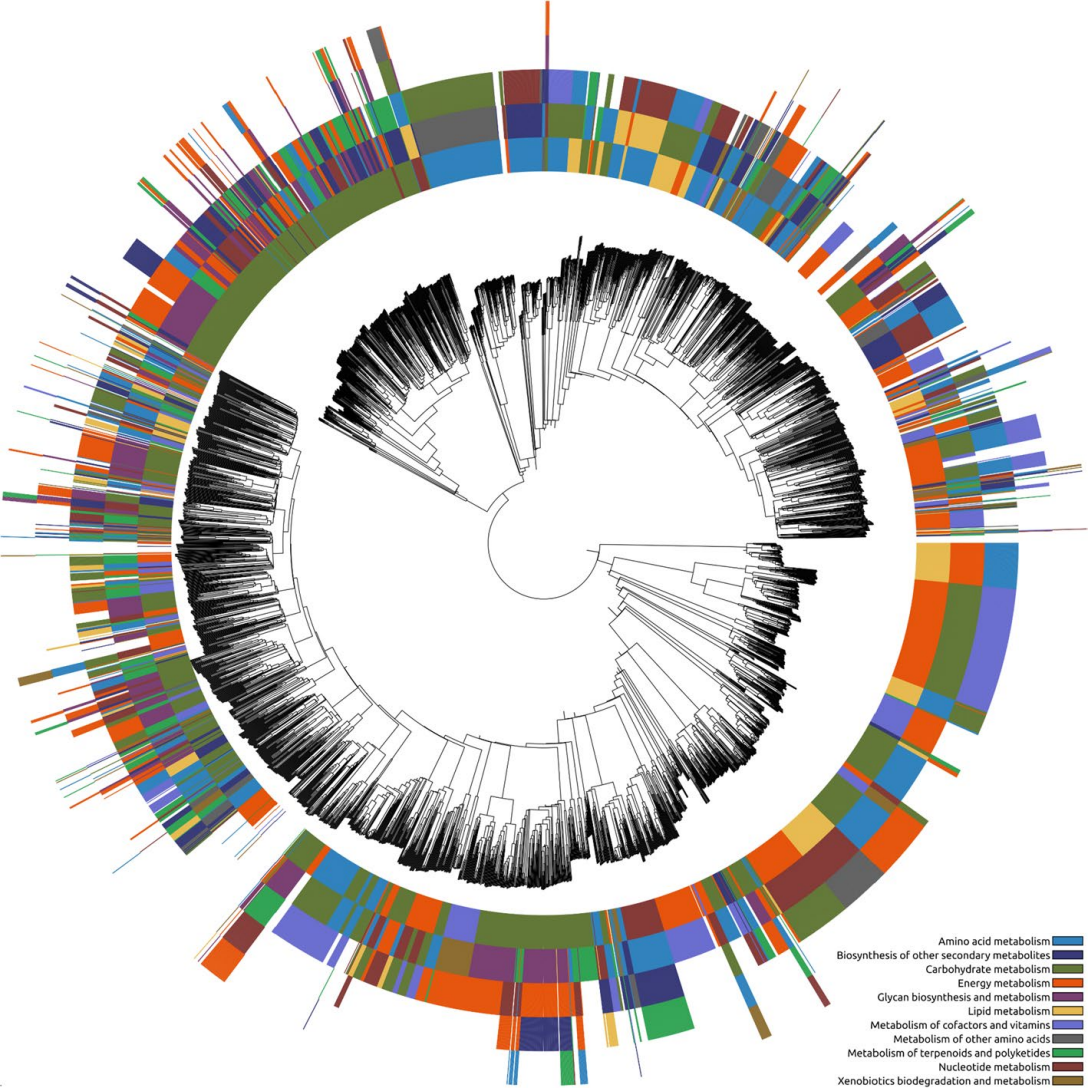
Phylogenetic tree of the protein sequences in the database paired with the metabolic categories of the compound(s) that bind to the TF

The 3507 sequences were aligned and assembled in a phylogenetic tree using Clustal Omega. The ete3 python library was used to produce the tree figures coupled with the categorical information. The chemical and metabolic categories previously determined were paired with each sequence in the tree producing the Figs. 7 and 8.

### Predictive model performance

**Fig. 9:**
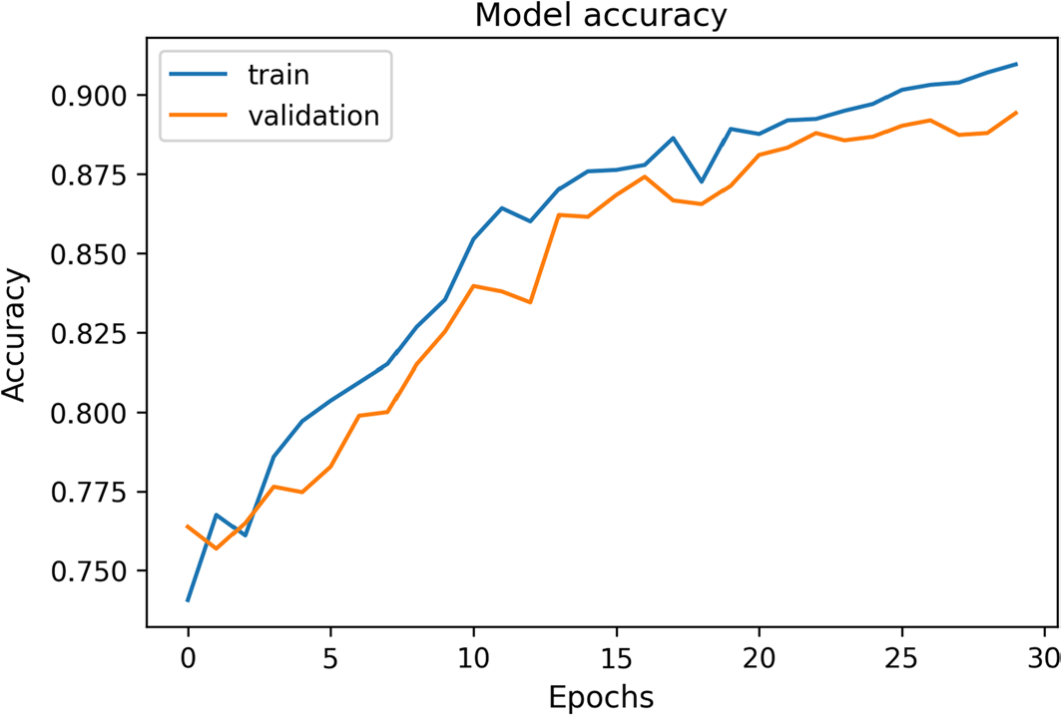
Example of accuracy curve for one of the validation processes of the final model

**Fig. 10:**
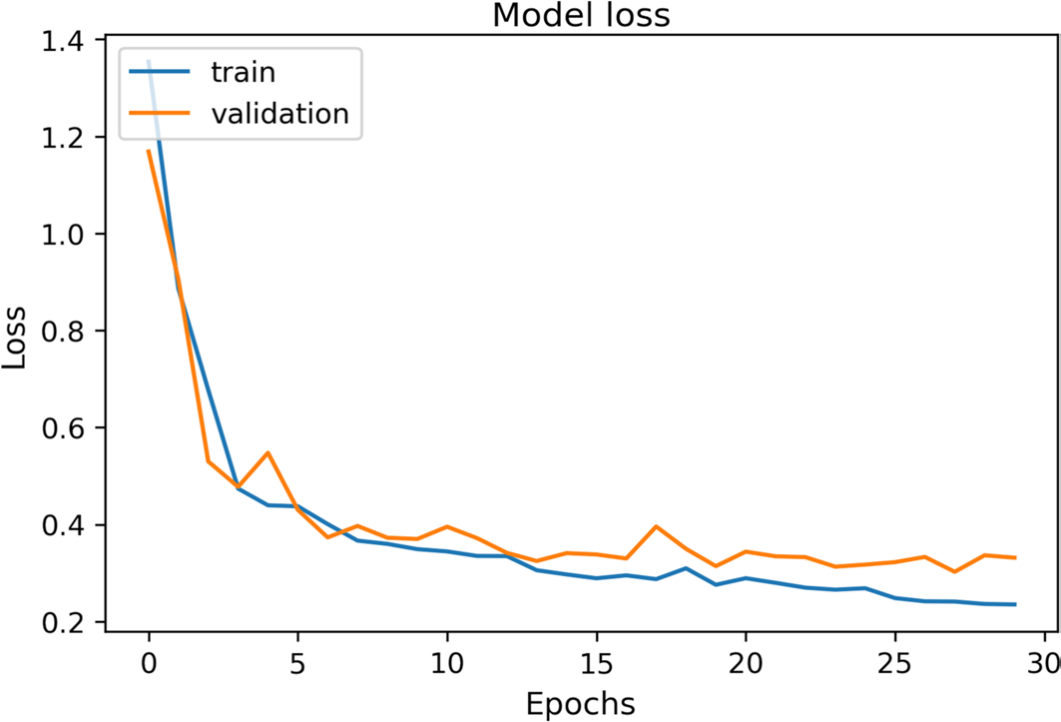
Example of loss curve for one of the validation processes of the final model

For the machine learning-based model shown in Fig. 2, loss and accuracy metrics were used during the validation process. Their evolution curves over the epochs of training during the last validation are shown in Figs. 9 and 10. The average loss value was 1.67 and the accuracy value was 80.7%.

The stabilization of both, accuracy and loss curves, and the evolution of the validation curve with respect to the train one, show that the number of epochs is enough to obtain acceptable results without overfitting.

**Fig. 11:**
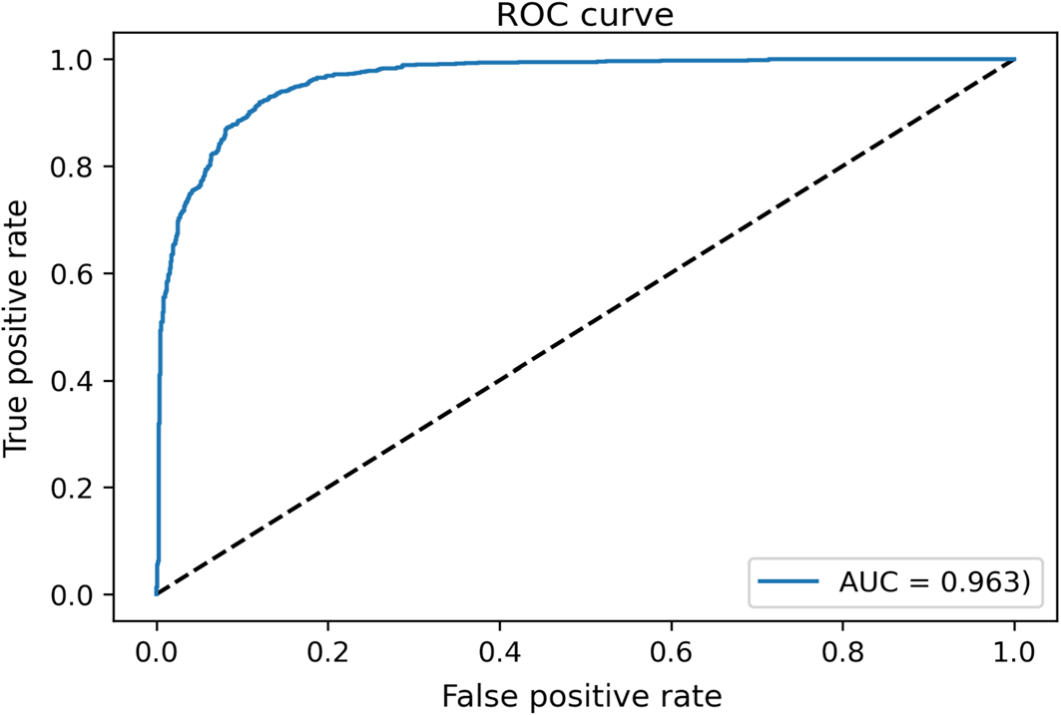
ROC curve resulting from test and its AUC value

After the validation, the actual model training was carried out. The scores obtained when predictions were made with the test data have been 0.3 of loss and 96,048% of accuracy. The ROC curve (Fig. 11) and the AUC value have also been obtained.

The evolution of the ROC curve and the AUC value associated led to the conclusion that the model performs reasonably well as a classifier between positive (there is affinity between the TF and the ligand) and negative (there is no affinity) cases.

Lastly, to compare the performance of each class (affinity between the TF and the molecule or not), a F1-score close to 0.9 has been obtained for both positive and negative cases. The similarity between the F1-score of the two groups demonstrates that the model is well balanced for predicting either the affinity between a TF and a molecule or the impossibility of using the TF to sense the molecule.

In order to verify the performance of our model, we have compared the results with other classification algorithms. These results are shown in Table 3 below:

**Table 3:** Performance comparison between different models

Model	Mean Accuracy (%)	Accuracy Std	Mean Loss
Original	96.048	0.490	0.292
SVM	88.581	0.706	0.711
RandomForest	95.496	0.408	1.551
Naive Bayes	76.002	0.453	8.649
Embedding	94.128	1.092	0.442

## Conclusions and future directions

In this study, we present two resources that may ease the biosensor design process and help researchers prototype biosensor circuits faster.

The first one is the Sensbio toolbox. By importing the algorithms into a notebook or another Python application or through the GUI-app, the system can suggest putative aTFs that may be able to detect a given input compound. The tool can also be used to determine the possible ligand molecule of a newly discovered TF sequence by homology to the database. The tool is available at https://bit.ly/3OF4msH.

Secondly, the ML model built in this study can be used to find extra TF-ligand interactions through a predictive system. Even if the results are promising, predictions of the ML-based model still lack enough specificity, as we are expecting to use this tool in order to refine the homology search. Future work will test other model architectures, including using the homology search results as additional input to the model.

Besides the improvement of ML-based predictions, the current dataset can be augmented with TF homologues in the positive dataset to improve further the prediction metrics. In the future, the ML model will be improved and integrated in the application. This could add an extra layer of certainty to trust the predicted TF-ligand interaction based on factors other than sequence or molecular similarity. An additional layer of information useful to the users may be the computation of structural-based scores for each TF-ligand pair from tools like molecular docking.

## Supplementary Information

**Additional file 1.** Complete molecular result output of five molecules (naringenin, pinocembrin, eucalyptol, luteolin and apigenin).

## Data Availability

The datasets used and analysed during the current study are available in the Zenodo repository 10.5281/zenodo.7432222.
